# Characteristics of asbestos fibers in lung tissue from occupational and environmental asbestos exposure of lung cancer patients in Busan, Korea

**DOI:** 10.1038/s41598-020-77291-9

**Published:** 2020-11-23

**Authors:** Hyun-Sung Jung, Eun-Kee Park, Jun-Seok Cha, Jae-Won Lee, Jong-Chun Lee, Jinyoung Jang, Suejin Kim, Chulho Oak, Deborah H. Yates, Hyunwook Kim

**Affiliations:** 1 Indoor Environment and Noise Research Division, National Institute of Environmental Research, Incheon, Korea; 2 Department of Public Health, Graduate School, The Catholic University of Korea, Seoul, Korea; 3 Department of Medical Humanities and Social Medicine, College of Medicine, Kosin University, Busan, Korea; 4 Climate Change and Disaster Management, Ministry of Health and Medical Services, Fiji, Suva, Fiji; 5 The Korean Association of Internal Medicine, Seoul, Republic of Korea; 6 Humidifier Disinfectant Health Center, National Institute of Environmental Research, Incheon, Korea; 7 Department of Internal Medicine, College of Medicine, Kosin University, Busan, Korea; 8 Department of Thoracic Medicine, St Vincent’s Hospital, Sydney, Australia; 9 Department of Preventive Medicine, College of Medicine, The Catholic University of Korea, 222 Banpo-daero Seocho-gu, Seoul, Korea

**Keywords:** Environmental sciences, Risk factors

## Abstract

The Asbestos Injury Relief Act in Korea requires that asbestos exposure be assessed through clinical examination and chest computed tomography (CT). However, a more specific measurement of asbestos characteristics in the lung tissue may be appropriate. We aimed to investigate the asbestos burden and characterize asbestos fibers in patients with lung cancer and ultimately assess the relationship between occupational and environmental asbestos exposure and lung cancer in Korea. We evaluated 37 lung cancer patients (LCPs) from Busan. The factors affecting asbestos burden in LCPs were analyzed using a multiple regression analysis. History of asbestos exposure (environmental/occupational), male sex, and old age were the main factors affecting asbestos burden in lung tissues of LCPs. These factors had an approximate 37% adjusted coefficient of determination. There was a significant difference in the length of asbestos fibers (4.06–37.6 µm vs. 4.26–91.7 µm) and aspect ratio (4.5–151.9 vs. 5.6–735.6) between those who were occupationally exposed to asbestos and those who were environmentally exposed (P < 0.01). Therefore, both environmental/occupational exposure to asbestos should be strongly managed to reduce the risk of lung cancer, and exposure should be assessed according to the characteristics of asbestos fibers in the lung tissue.

## Introduction

Although smoking is the primary risk factor for lung cancer, asbestos exposure is also an acknowledged risk factor for this malignancy^1–3^. There is adequate evidence supporting that all six types of asbestos are carcinogenic and can cause mesothelioma and lung cancer, laryngeal cancer, and ovarian cancer in humans^4^. In relation to this, there is an increasing concern regarding the role of environmental asbestos exposure in the causation of asbestos-related lung cancer^5,6^. In Korea, the incidence of pulmonary diseases due to environmental asbestos exposure has recently emerged as a social issue, highlighting the need for more investigations detecting asbestos levels in the air and soil as well as addressing public health concerns related to asbestos exposure since Korea banned asbestos use in 2009^7^. The Asbestos Injury Relief Act (KAIRA) in Korea stipulates the compensation criteria for asbestos-related diseases due to environmental exposure to asbestos. The KAIRA requires clinical examination and a chest computed tomography (CT) scan, with the success of the claim based on the results of both of these assessments^8^.

The development of asbestos-related diseases depends on the dose and form of asbestos and morphological characteristics of the fibers, including length and diameter, that affect the durability or persistence of asbestos fibers in the lung^9^. Stanton reported that long (> 8 µm) and thin (< 0.25 µm) mineral fibers were strongly carcinogenic and induced the development of pleural mesothelioma in rats^10^. However, Suzuki and Yuen^11^ reported that most asbestos fibers detected in the lung and mesothelial tissues were shorter than those of Stanton’s dimensions, with only 4.0% satisfying Stanton’s dimensions. For lung cancer, a significantly positive correlation has been found between long, thin fiber dimensions and an increase in lung cancer incidence^12–14^. Lung cancer has a stronger association with exposure to longer and thinner fibers, and models for these fibers also support such findings^13^. Asbestos fiber burden in the lung provides useful information for evaluation of past exposure levels and can thus be an option of providing evidence in determining the relationship between asbestos and related diseases^15,16^.

KAIRA has set two criteria for establishing the occurrence of primary lung cancer from asbestos exposure, with consideration of the incubation period from asbestos exposure to the outbreak and amount of exposure force, among other factors. First, primary lung cancer is diagnosed through either biopsy/pathological examination or clinical/imaging judgment when biopsy/pathological examination cannot be performed. Second, the diagnosis of primary lung cancer is medically established if one of the following conditions is fulfilled: (1) the morbid type is progressive or early form according to the pathology of asbestos lung disease, (2) there is a pleural plaque caused by asbestos, (3) there are more than 5,000 asbestos fibers per gram of dry lung weight, (4) there are more than 5,000,000 asbestos fibers with a length of more than 1 µm per 1 g of dry lung weight, (5) there are at least 2,000,000 asbestos fibers with a length of more than 5 µm per 1 g of dry lung weight, and (6) there are more than 5 asbestos bodies per 1 ml of bronchoalveolar cleaning solution. However, determining the characteristics of asbestos fibers in the lung may represent a more quantitative estimation of asbestos exposure history^17^. Previous studies on asbestos exposure in patients with pulmonary disease in Korea have been limited to autopsy subjects^18–20^. Therefore, analyses of asbestos in the lung tissues and the data on characteristics of those exposed to asbestos are limited.

The purpose of this study was to investigate the asbestos burden and characterize asbestos fibers in patients with lung cancer to obtain basic data that can be used for asbestos control policies in Korea. We hypothesized that asbestos burden, as well as asbestos characteristics (length, width, aspect ratio), would be associated with the development of lung cancer in patients exposed to asbestos.

## Results

### Patient characteristics

There were 25 and 12 male and female patients, respectively, and the mean patient age was 61.5 (range 28–79) years. Overall, 21 patients were aged < 65 years, while 16 patients were aged ≥ 65 years, and 26 and 11 patients were smokers and nonsmokers, respectively. In total, 6 patients (16.2%) had self-reported occupational exposure history to asbestos, and 31 patients (83.8%) reported that asbestos factories were located within a 5-km radius of their residence or their living environment, confirming environmental asbestos exposures. The patient characteristics are presented in Table 1.

**Table 1:** Clinicodemographic patient characteristics.

Sex	N	Age (years)		Smoking		History of asbestos exposure		Wet (g)		Dry (g)		D/W	
		Mean	Range	Yes	No	Environmental	Occupational	Mean	Range	Mean	Range	Mean	Range
Male	25	61.9	28–76	25	–	19	6	1.96	0.56–3.39	0.22	0.07–0.44	11.8	7.6–20.4
Female	12	60.9	30–79	1	11	12	–	2.25	1.28–3.17	0.26	0.14–0.42	11.5	7.6–13.9
Total	37	61.5	28–79	26	11	31	6	2.05	0.56–3.39	0.23	0.07–0.44	11.7	7.6–20.4

Of the six people surveyed for their occupational exposure to asbestos, exposure history was from (1) work at an asbestos mine, (2) work at an asbestos insulation-handling site of a dockyard for 13 years, (3) boiler maintenance work using asbestos cloth occasionally, (4) pigpen construction work using asbestos slates for approximately 6–7 years, (5) insulation work using glass wools and asbestos cloths at a dockyard for 30 years, and (6) work at an asbestos cement board manufacturing company.

### Asbestos burden in lung tissue

The detection limit varies depending on the dry tissue weight and the number of grid openings scanned. In this study, the detection limit ranged from 0.021 to 0.202 million fiber/g of dry lung tissue. The results of TEM analyses of asbestos burden in lung tissues are presented in Tables 2 and 3. The asbestos burden was significantly different between LCPs aged under 65 years and ≥ 65 years (0.049 × 10^6^ fiber/g vs. 0.094 × 10^6^ fiber/g, P < 0.05), but not between male and female patients (0.070 × 10^6^ fiber/g vs. 0.064 × 10^6^ fiber/g). There was also no significant difference in asbestos burden between 26 smokers and 11 nonsmokers (0.067 × 10^6^ fiber/g vs. 0.076 × 10^6^ fiber/g) and between the 23 EA-LCP and the 6 OA-LCP (0.059 × 10^6^ fiber/g vs. 0.120 × 10^6^ fiber/g) (Table 2).

**Table 2:** Geometric mean values of asbestos burden in lung tissues by sex, smoking, and history of asbestos exposure.

Variable	n	Asbestos burden (fibers/g of dry lung tissue)			P-value
		Detection, n	Range	GM [GSD]	
**Sex**					
Male	25	21	0.024 × 10^6^–0.41 × 10^6^	0.070 × 10^6^ [2.29]	0.8108
Female	12	8	0.021 × 10^6^–0.59 × 10^6^	0.064 × 10^6^ [2.89]	
**Age, years**					
< 65	21	14	0.021 × 10^6^–0.23 × 10^6^	0.049 × 10^6^ [2.15]	0.0456
≥ 65	16	15	0.033 × 10^6^–0.59 × 10^6^	0.094 × 10^6^ [2.43]	
**Smoking**					
Yes	26	22	0.021 × 10^6^–0.41 × 10^6^	0.067 × 10^6^ [2.34]	0.7342
No	11	7	0.025 × 10^6^–0.59 × 10^6^	0.076 × 10^6^ [2.81]	
**History of asbestos exposure**					
Environmental	31	23	0.021 × 10^6^–0.59 × 10^6^	0.059 × 10^6^ [2.41]	0.0821
Occupational	6	6	0.046 × 10^6^–0.27 × 10^6^	0.120 × 10^6^ [1.96]	

*GM* geometric mean concentrations; *GSD* geometric standard deviation.

**Table 3:** ANOVA results of asbestos burden in lung tissues by TEM examination.

Variable			Asbestos burden (fibers/g of dry lung tissue)			p-value
History of exposure	Sex	n	Detection, n	Range	GM [GSD]	
Environmental	Male	19	15	0.024 × 10^6^–0.41 × 10^6^	0.057 × 10^6^ [2.56] ^a^	0.2154
	Female	12	8	0.021 × 10^6^–0.59 × 10^6^	0.064 × 10^6^ [2.89] ^a^	
Occupational	Male	6	6	0.046 × 10^6^–0.27 × 10^6^	0.120 × 10^6^ [1.96] ^a^	
Total		37	29	0.021 × 10^6^–0.59 × 10^6^	0.069 × 10^6^ [2.41]	

*GM* geometric mean concentrations; *GSD* geometric standard deviation.

The asbestos burden was 0.057 × 10^6^ fiber/g for the 15 male EA-LCPs, 0.064 × 10^6^ fiber/g for the 8 female EA-LCPs, and 0.120 × 10^6^ fiber/g for the 6 male OA-LCP (Table 3). There was no significant difference in asbestos concentrations according to sex in those who were environmentally exposed to asbestos.

### Analysis of the factors affecting asbestos burden in lung tissue

Multiple regression analysis showed that the history of asbestos exposure (environmental/ occupational), sex (male), and age (61–70, ≥ 71 years) were the main factors affecting the asbestos burden in lung tissue of LCPs, and the adjusted coefficient of determination of these factors was 37.1% (Table 4). The variance inflation factor was less than 10 in the history of asbestos exposure, sex, and age, indicating that multi-collinearity did not exist between factors.

**Table 4:** Results of multiple regression analysis of affecting factors of asbestos burden.

Variables	Standard	Variables	Parameter estimates	Standard error	Standardized coefficients	P-value
History of exposure	Environmental exposure	Occupational exposure	0.37465	0.05693	0.55473	< 0.0001
Sex	Female	Male	− 0.11433	0.05325	− 0.18023	0.0339
Age, years	≤ 50	51–60	− 0.07262	0.11800	− 0.08748	0.5395
		61–70	− 0.39807	0.10846	− 0.67838	0.0004
		≥ 71	− 0.26949	0.11334	− 0.41109	0.0190
	*F*			15.50		
	p-value			< 0.0001		
	Adjusted R^2^			0.3709		

Regarding the standardized coefficient of the asbestos burden in the lung tissue, history of occupational exposure (0.55473, P < 0.0001) showed a higher association with the asbestos burden in the lung tissue than history of environmental exposure. In addition, an older age (> 61 years) and the male sex showed stronger association with the asbestos burden (P < 0.05).

### Asbestos fiber characterization in lung tissue

The morphology, chemical components, and selected area electron diffraction of asbestos fibers in the lung tissues detected through TEM analysis are presented in Table 5. The types of asbestos detected in the lung tissues were chrysotile, amosite, crocidolite, tremolite, and actinolite, and we were able to assess the shape of the bundles in the enlarged image. Chrysotile was shaped in a bundle, while the remaining asbestos types were shaped as a single fiber (Table 6).

**Table 5:**
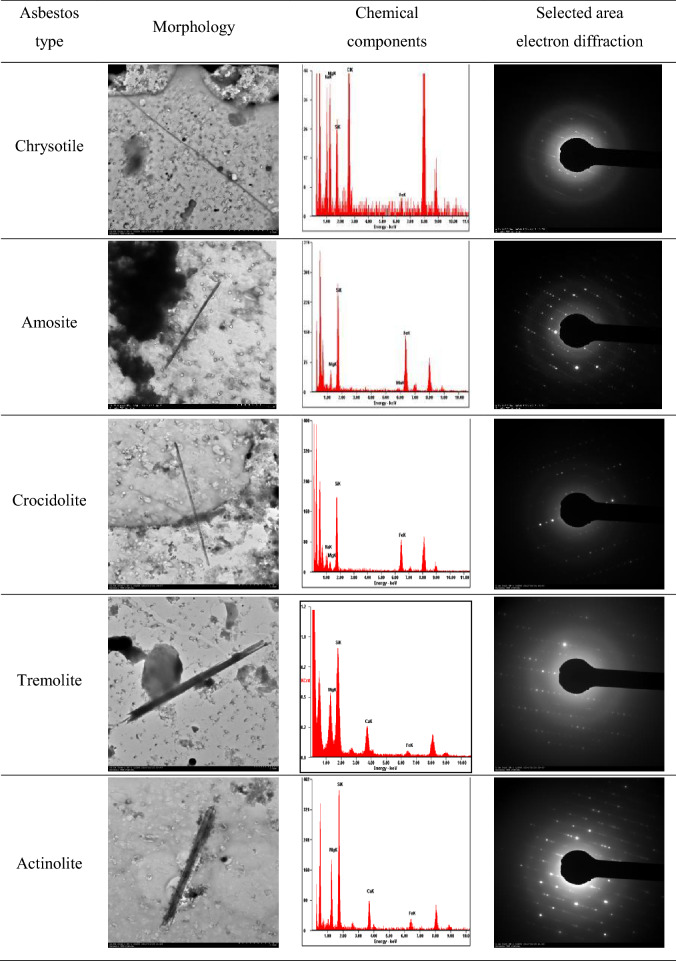
Morphology, chemical components (via energy-dispersive x-ray spectrometer), and selected area electron diffraction of detected asbestos fibers in lung tissue.

**Table 6:**
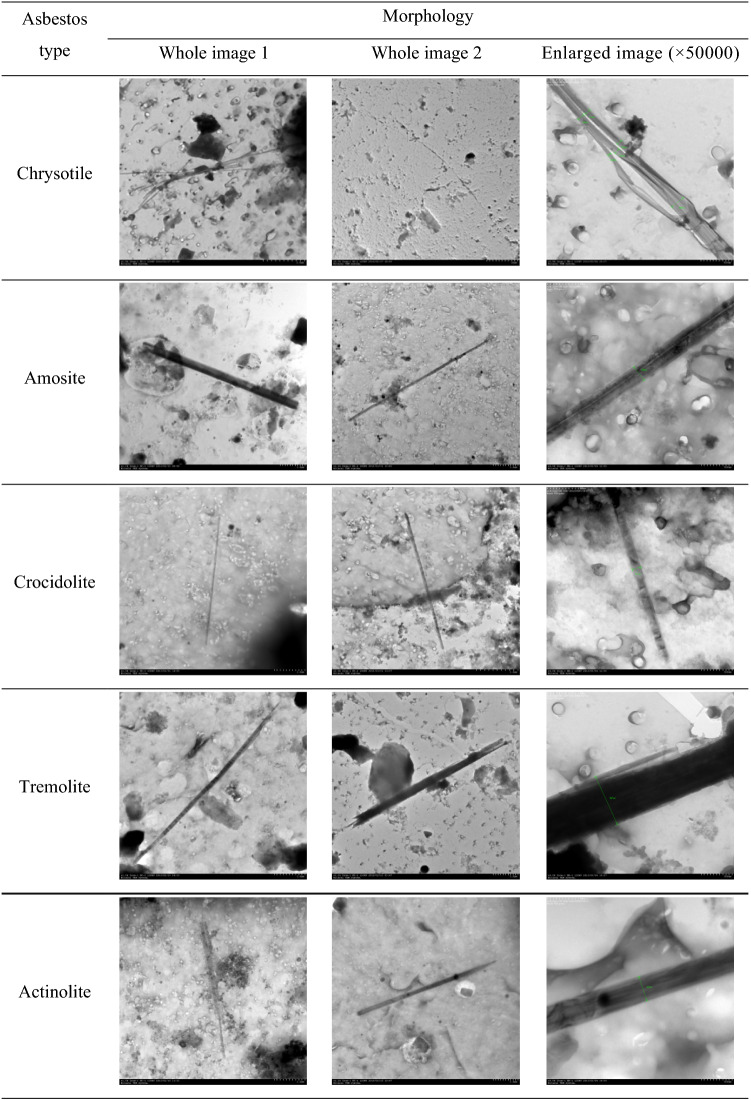
Comparison of morphology of detected asbestos fibers in lung tissue between a whole image and an enlarged image.

**Figure 1:**
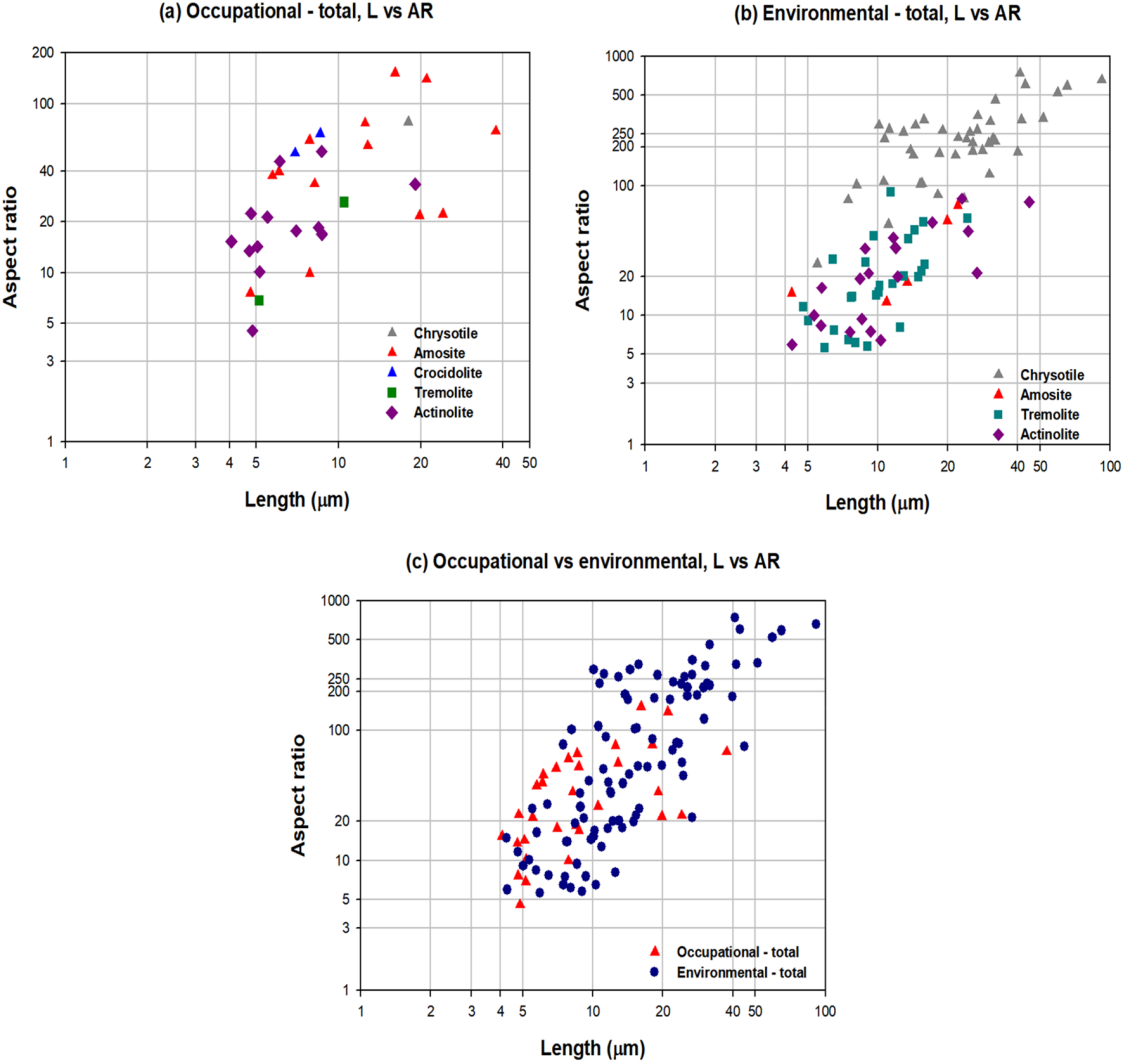
Distribution of asbestos fiber length and aspect ratio according to type of asbestos exposure. (**a**) Occupational—total, L vs. AR. (**b**) Environmental—total, L vs. AR. (**c**) Occupational vs. environmental, L vs. AR.

The following asbestos fibers were detected in the lung tissue of 29 LCPs: 42 chrysotile, 5 amosite, 26 tremolite, and 20 actinolite (n = 93) in the 23 EA-LCPs and 1 chrysotile, 13 amosite, 2 crocidolite, 2 tremolite, and 13 actinolite (n = 31) in the 6 OA-LCPs. In the OA-LCPs, the length of the asbestos fiber ranged from 4.06 µm to 37.6 µm, with aspect ratios of approximately higher than 4. In the EA-LCPs, the length of the asbestos fibers ranged from 4.26 µm to 91.7 µm, with aspect ratios of higher than 5. There were no statistically significant differences in the individual lengths of the asbestos types between the EA-LCPs and the OA-LCPs, except those for actinolite (Table 7, Fig. 1). Meanwhile, the overall asbestos fiber length was significantly different between EA-LCPs and OA-LCPs (P < 0.001) (Table 7).

**Table 7:** Morphological characteristics (length, diameter, and aspect ratio) of the detected fibers by asbestos type.

History of asbestos exposure	Environmental							Occupationa							p-value		
	n	Length (μm)		Diameter (μm)		Aspect ratio		n	Length (μm)		Diameter (μm)		Aspect ratio				
Type		GM [GSD]	Range	GM [GSD]	Range	GM [GSD]	Range		GM [GSD]	Range	GM [GSD]	Range	GM [GSD]	Range	L	D	AR
Chrysotile	42	21.83 [1.85]	5.49–91.7	0.105 [1.67]	0.035–0.298	208.45 [2.00]	24.84–735.61	1	18.00	–	0.232	–	77.59	–	0.822	0.822	0.822
Amosite	5	12.23 [1.93]	4.26–22.1	0.466 [1.67]	0.288–0.864	26.25 [2.20]	12.62–70.16	13	11.74 [1.89]	4.76–37.6	0.293 [2.31]	0.106–1.090	40.02 [2.47]	7.57–151.89	0.758	0.132	0.167
Crocidolite	ND	–	–	–	–	–	–	2	7.70 [1.16]	6.93–8.56	0.132 [1.04]	0.129–0.136	58.15 [1.21]	50.96–66.36	–	–	–
Tremolite	26	9.99 [1.49]	4.77–24.3	0.564 [1.84]	0.128–1.570	17.71 [2.15]	5.58–89.06	2	7.34 [1.66]	5.13–10.5	0.549 [1.56]	0.402–0.751	13.36 [2.58]	6.83–26.12	0.304	0.954	0.624
Actinolite	20	11.07 [2.13]	4.29–44.9	0.557 [1.71]	0.269–1.600	19.86 [2.29]	5.93–79.58	13	6.45 [1.51]	4.06–19.1	0.349 [1.75]	0.134–1.070	18.46 [1.89]	4.53–52.35	0.005	0.023	0.789
Total	93	14.69 [1.94]	4.26–91.7	0.260 [2.69]	0.035–1.600	56.45 [4.07]	5.58–735.61	31	8.74 [1.79]	4.06–37.6	0.310 [2.02]	0.106–1.090	28.21 [2.39]	4.53–151.89	< 0.0001	0.287	0.002

*AR* aspect ratio; *D* diameter; *GM* geometric mean concentrations; *GSD* geometric standard deviation; *L* length.

**Figure 2:**
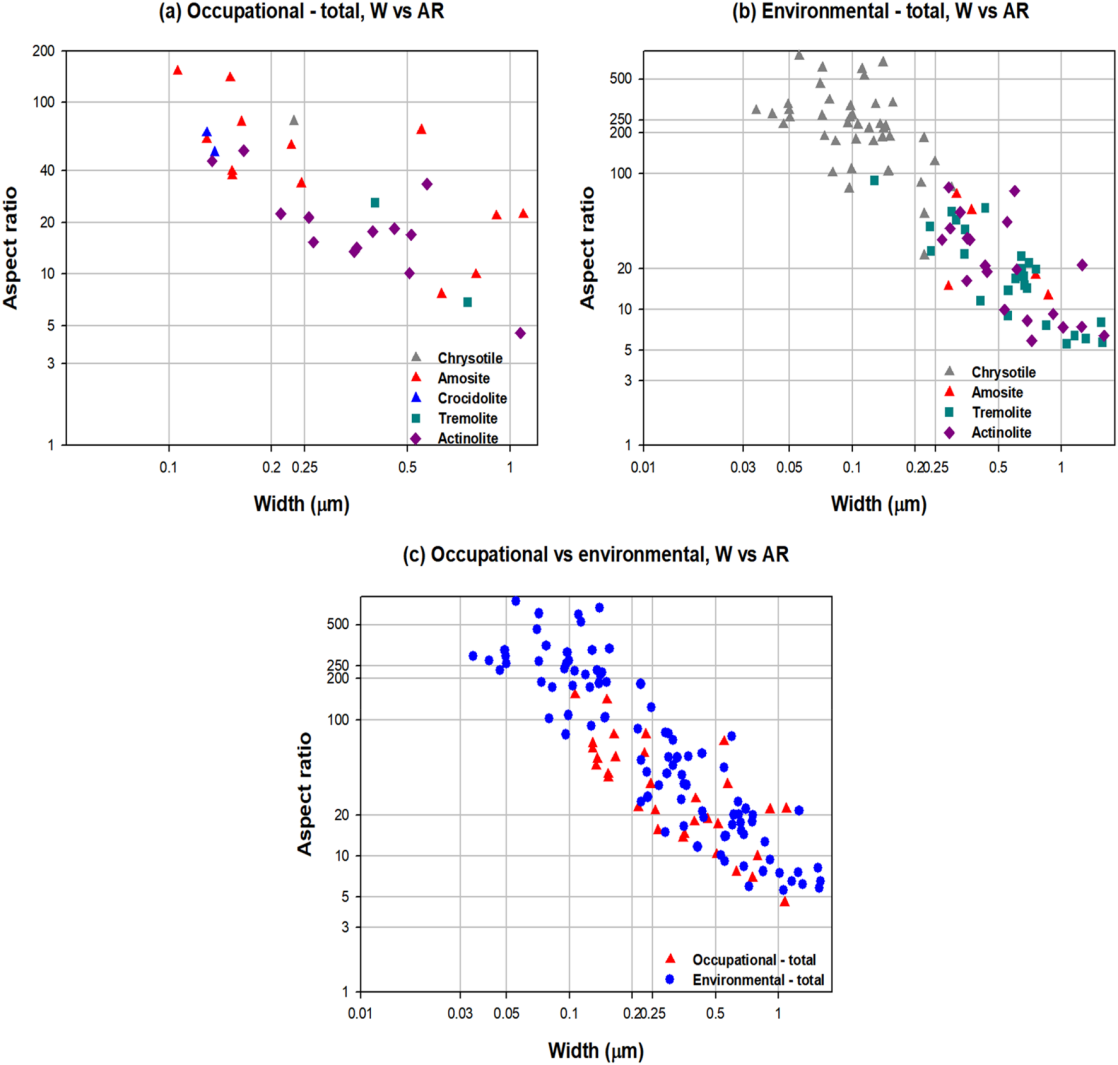
Distribution of asbestos fiber width and aspect ratio according to type of asbestos exposure. (**a**) Occupational—total, W vs. AR. (**b**) Environmental—total, W vs. AR. (**c**) Occupational vs. environmental, W vs. AR.

With respect to asbestos fiber diameter, it ranged from 0.106 µm to 1.090 µm, with aspect ratios of approximately higher than over 4 in OA-LCPs. Meanwhile, it ranged from 0.035 µm to 1.600 µm with aspect ratios of higher than 5 in EA-LCPs, with no significant difference (Table 5, Fig. 2). TEM analysis showed no trace of talc in the lung tissues although talc can be contaminated by asbestos fibers, which is in turn strongly related to the development of lung cancer.

## Discussion

The current study evaluated the asbestos burden and characteristics of asbestos fibers detected in the lung tissues of 37 LCPs who self-reported a history of asbestos exposure. There was no significant difference in the asbestos burden according to history of asbestos exposure (occupational vs. environmental). However, the asbestos length and aspect ratio were significantly different between OA-LCPs and EA-LCPs. History of asbestos exposure (environmental/occupational), sex, and age were the main factors affecting the asbestos burden in lung tissue of LCP.

There have been several previous studies examining the total asbestos burden of patients with asbestos-related diseases in Korea, but these have mainly been autopsy studies conducted before the changes in regulation on January 1, 2011. Yu et al.^20^ found an asbestos burden of 0.26 × 10^6^ fiber/g of dry lung tissue in 20 autopsy subjects, while Lim et al.^19^ reported an asbestos burden of 0.09 × 10^6^ fiber/g in 22 autopsy subjects. Han et al.^18^ reported a burden of 0.22 × 10^6^ fiber/g in 36 normal subjects and 0.19 × 10^6^ fiber/g in 38 subjects with lung cancer. In addition to Korean studies, 3 Japanese studies reported the mean asbestos burden as fibers per gram of dry lung tissue. Sakai et al.^21^ reported an asbestos burden of 2.11 × 10^6^ fiber/g in 25 men with an occupational history of asbestos exposure and 4.41 × 10^6^ fiber/g in 19 men and 1.38 × 10^6^ fiber/g in 25 women without an occupational history of asbestos exposure. In a later study, Sakai et al.^22^ reported an asbestos burden of 22.0 × 10^6^ fiber/g in 16 patients with mesothelioma and 2.24 × 10^6^ fiber/g in 16 control patients. Sakai et al.^23^ reported an asbestos burden of 4.49 × 10^6^ fiber/g in 13 men with a history of occupational asbestos exposure.

With respect to environmental exposure to asbestos, Sakai et al.^21^ reported an asbestos burden of 1.67 × 10^6^ fiber/g in 36 of 53 urban residents without occupational asbestos exposure compared with 5.82 × 10^6^ fiber/g in 13 urban residents with a history of asbestos exposure in Japan. In a study of 49 urban residents (31 men and 18 women) living > 70 km from the area (Aichi Shinshiro and Minami-Kitashitara regions) where serpentine asbestos is found, Sakai et al.^23^ reported that 18 male and 18 female patients without a history of occupational asbestos exposure had asbestos burdens of 2.05 × 10^6^ fiber/g and 1.38 × 10^6^ fiber/g, respectively. Our findings are consistent with other lung-burden studies showing a gradation of fiber burdens from occupationally exposed to domestically exposed persons.

The asbestos detected in our study varied in type, consistent with that in the literature^10,17–23^. Fiber diameter affects airborne fiber penetration and along the lung airways and thus initial deposition patterns. Toxicological data have shown that lung cancer is most strongly related to exposure to fibers with diameter < 0.25 µm. Our findings of the relationship between lung cancer and fiber diameter are similar to those of Adib et al.^24^ and Loomis et al.^12,25^. Moreover, our results are similar to those of Lippmann, who reported that the risk of lung cancer and asbestosis is related to exposure to fibers measuring > 0.15 µm in diameter^26^. However, it was not possible to draw conclusions on the risks of associations between lung cancer and asbestos diameter in terms of policy.

In an epidemiological and exposure-evaluation study of patients grouped by environmental exposure, Stayner et al.^13^ demonstrated a stronger association between lung cancer and long, thin fibers (length, > 10 µm; diameter, < 0.25 µm) than with short (< 5 µm) or thick (> 3.0 µm) fibers. The geometric mean of the asbestos burden (0.069 × 10^6^ fiber/g) due to history of asbestos exposure (occupational/environmental) in the current study was lower than that in previous studies^18–23^. Specifically, 79% of detected asbestos fibers in previous studies were > 5 µm in length^19–21^, whereas in the present study, no asbestos fiber < 4 µm in length was detected. This discrepancy could be possibly due to regional differences in exposures and differences in the pre-treatment methods of the lung tissue. Previous studies^18–20^ in Korea processed lung tissues by pretreating them low-temperature ashing following the method of Sakai et al.^21^, whereas the present study used the digestion method. Gylseth et al.^27^ and Rogers^28^ pointed out that ashing in high and low temperatures fragments long fibers into short ones, thus resulting in an increased asbestos concentration. Therefore, the geometric mean of length of the detected asbestos fibers in our study differed from those of previous studies. In addition, the asbestos burden can differ between studies because of the difference in the analytical sensitivity. However, although the digestion method^27^ is complicated by issues of incomplete removal of organic materials, which could negatively affect electron microscopy analysis, the digestion method in the present study facilitated TEM analysis. Therefore, this study is expected to be a complementary important basis for accurately assessing asbestos damage by calculating asbestos exposure estimates through asbestos measurement in the lung tissue. In addition, counting asbestos fiber could be a proper method during a process of compensation for asbestos-related diseases, if data on asbestos exposure history are insufficient.

The strength of this study was that the characteristics of asbestos, such as the shape and length, as it was inhaled into the lungs of LCPs were identified through the new digestion pre-treatment method. In addition, TEM analyses were conducted with a lower analytical sensitivity than those in previous studies, and thus a more accurate asbestos burden was also assessed for LCPs with environmental exposure. However, our study also has some limitations. The number of patients was small, and the patients were all from the Busan region. Because asbestos exposure is not the major cause of lung cancer, the rate of asbestos detection in lung cancer is likely to be significantly lower than that in mesothelioma, where asbestos is the most likely single cause. Further studies on asbestos burden by occupational and environmental asbestos exposure histories need to be performed in LCPs from different regions to determine whether there is a correlation between the regions and accumulation of pulmonary asbestos burden, with consideration of naturally occurring asbestos, workplace, rural or urban areas.

In conclusion, the geometric mean asbestos burden of EA-LCPs was not significantly different compared with that of OA-LCPs, but it was more than twice as low. History of asbestos exposure was the most relevant factor affecting asbestos burden, and there was a statistically significant difference in length and aspect ratio in detected asbestos fibers between EA-LCPs and OA-LCPs. Overall, both environmental and occupational asbestos exposure and the characteristics of asbestos fibers (length, aspect ratio) were risk factors of lung cancer. Therefore, not only occupational exposure, but also environmental exposure to asbestos should be managed to reduce the risk of lung cancer.

## Methods

### Study design and population

This study was approved by the Institutional Review Board of Kosin University Gospel Hospital (IRB No. 12–039) and was conducted in accordance with the relevant guidelines and regulations. All patients provided informed consent.

**Figure 3:**
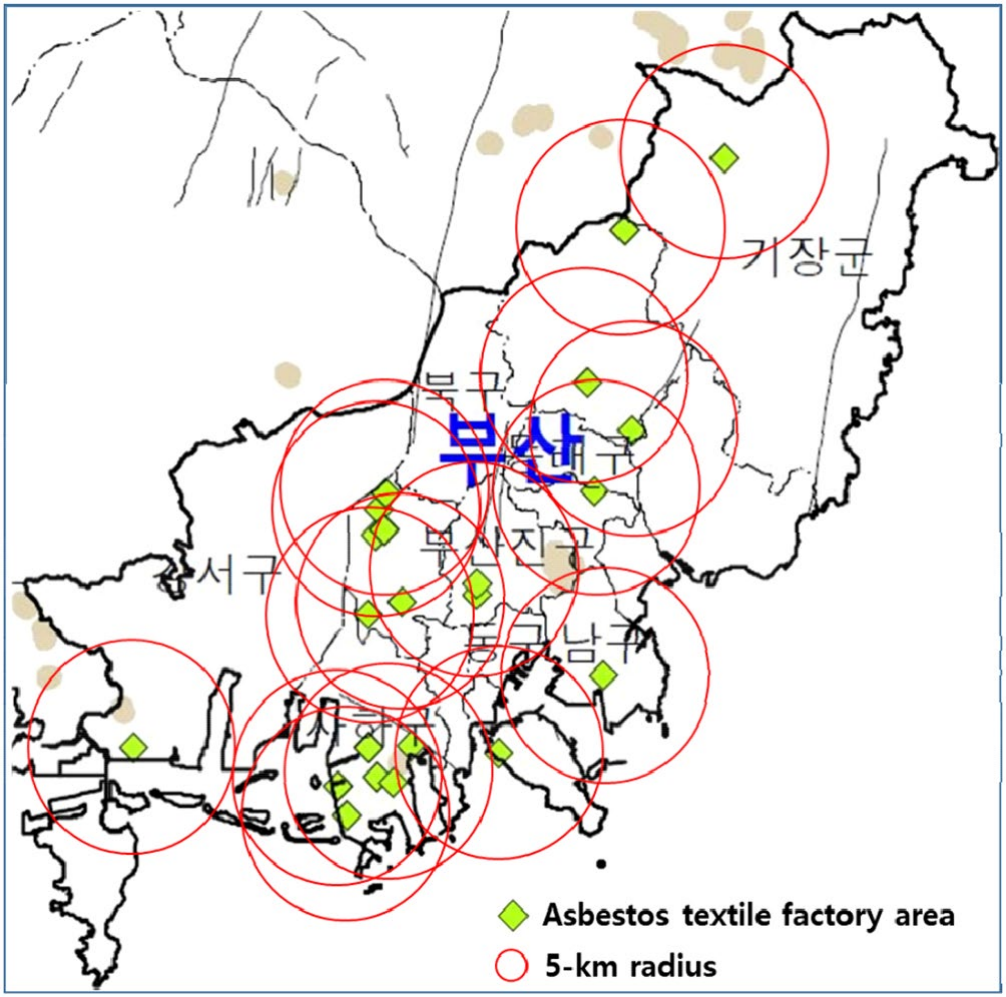
Map of asbestos textile factories in Busan. The green dots represent the location information of 22 asbestos factories. The red circle represents an area within a 5 km radius of the asbestos plant. This map was created using MapInfo Pro software (version 12.0, https://www.pitneybowes.com/us/location-intelligence/geographic-information-systems/mapinfo-pro.html) and edited by Hyun-Sung Jung. The base-map image, contour lines, topographic features are based on the regional geological map of naturally occurring asbestos. This is public data which were provided by “Asbestos Management Comprehensive Information Network” (https://asbestos.me.go.kr), Ministry of Environment of Korea (available from, accessed 13 November 2019).

The number of subjects was calculated using the GPOWER Samples Calculation Program (Faual et al. 2009). For multiple regression analysis, a statistically significant level of 0.05, power of 0.8, effect size level of 0.35, and three independent variables (i.e., history of exposure, sex, and age) were analyzed. The calculated target sample size was 36 people. Among general lung cancer patients who were scheduled for pneumonectomy within 6 months after the beginning of the study investigation, we randomly selected 37 lung cancer patients who agreed to participate as study subjects. Busan was selected as the target area for investigation because it used to have 22 asbestos textile factories, and raw materials of asbestos were imported until the early 2000s, with the main type being chrysotile that was used in asbestos textile factories (Fig. 3). Asbestos history was evaluated using a 16-item questionnaire (13 questions about occupational exposure and 3 questions on environmental exposure). Occupational exposure was assessed using the following 13 questions: (1) Have you ever worked in a Seoul subway station in 1980? (2) Have you ever worked in a slate manufacturing factory? (3) Have you ever been a firefighter and have been exposed to collapsed debris for at least a year? (4) Have you ever worked at a building demolition site? (5) Have you ever worked in an asbestos cement factory? (6) Have you ever worked in a brake pad or lining manufacturing plant? (7) Have you ever worked in a shipyard that handles asbestos insulation? (8) Have you ever worked in an asbestos mine? (9) Have you ever worked at an asbestos textile factory? (10) Have you ever worked in an asbestos tile manufacturing plant? (11) Have you ever worked in an asbestos-containing powder manufacturing plant? (12) Have you ever worked as an asbestos insulation worker? (13) Have you ever worked in an asbestos paint manufacturing plant? Meanwhile, environmental exposure was assessed according to the following three conditions: (1) whether the family members living in the same household had been involved in handling asbestos; (2) high exposure to dust while staying near a construction site for a certain period of time; and (3) existence of a factory handling asbestos within a 5-km radius of the residence or the living environment.

### Collection and preparation of lung tissue

Lung tissue specimens were obtained through pneumonectomy. Lung tumor regions and normal regions at the same site were identified pathologically, and a maximum of 5 g of the normal lung tissue was excised, fixed in 10% formalin solution, and preserved. The collected lung samples were divided into 3 blocks per sample and preserved in 10% formalin solution. To calculate the ratio of dried solid matter (%), a part of each biopsy sample was dried in a hood at room temperature (22 °C) for 7 days and dried again at 60 °C in an oven for 24 h. The final weights were then measured to calculate the ratio of dry to wet weight (D/W), which was used to calculate the asbestos burden of the lung tissues (fiber/dry g).

To prevent fat from interfering with asbestos analysis, a defatting solution was prepared by mixing ethanol filtered with a polycarbonate (PC) filter and ether in a 3:1 ratio. The defatting solution (25 mL) and sample tissues were collected in a capped 50-mL centrifuge tube, treated in an ultrasonic bath for approximately 1 min, incubated in a fume hood for approximately 2 h, and then centrifuged at 2600 rpm for 10 min at 4 °C, followed by removal of the supernatant. When an excess amount of debris was produced during the defatting process, this step was repeated two more times, and 10 mL of 100% ether was added to the remaining biopsy sample in the tube, followed by incubation in a fume hood for 10 min, centrifugation, and removal of the supernatant. Residual ether in the remaining biopsy sample was evaporated by loosening the tube cap and incubating in a fume hood for 15 min–1 h, depending on the sample.

**Figure 4:**
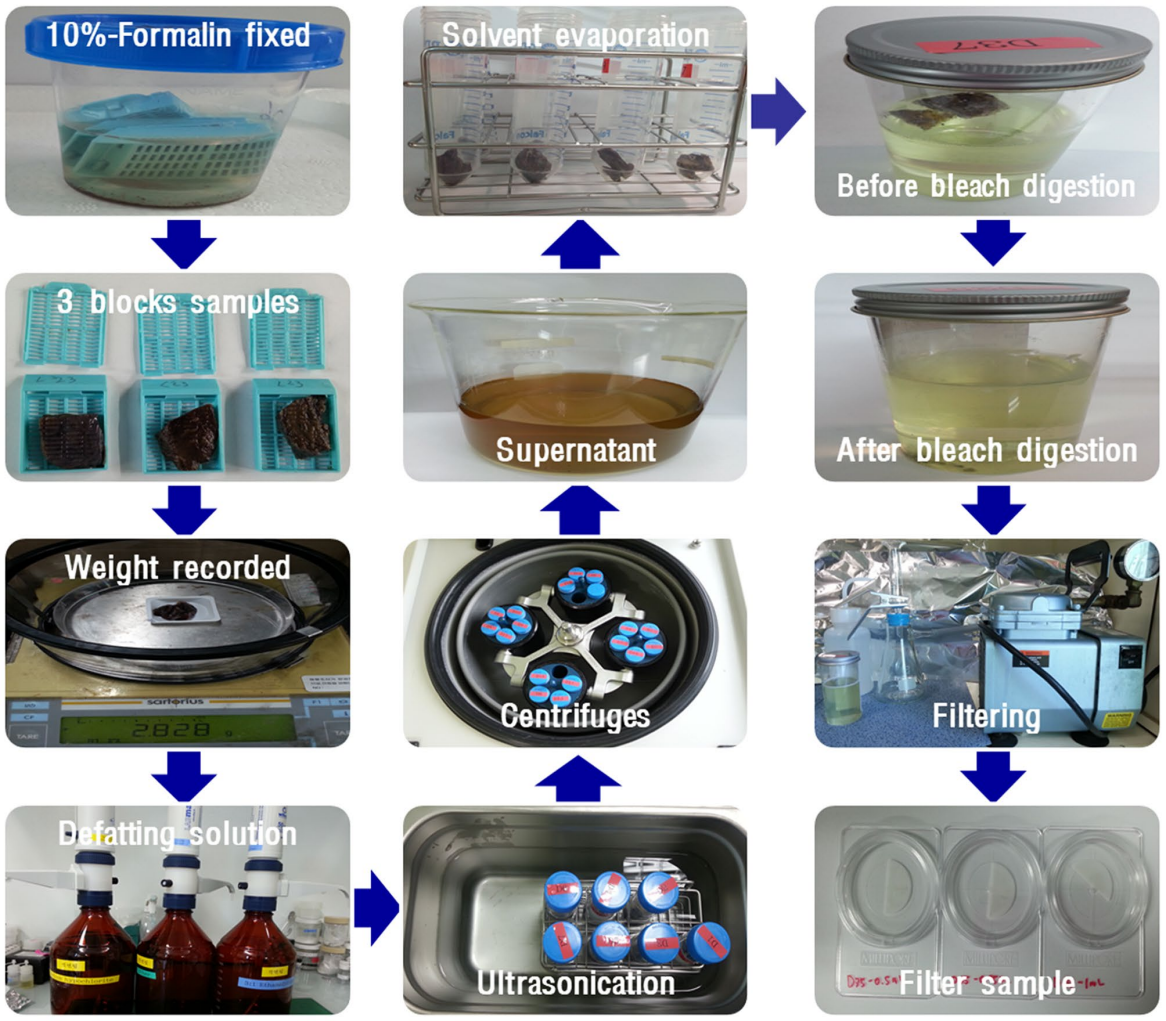
Pretreatment process.

The de-fatted biopsy sample and 100 mL of 5% sodium hypochlorite were mixed in a 150 mL container, and then bleach digestion was performed. The sample container was incubated at room temperature until the lung tissues were completely digested by the bleach. After shaking the container to evenly spread the bleach-digested biopsy particles in 5% sodium hypochlorite solution (100 mL), 1 mL of the lung bleach-digested solution was collected from the middle of the container and filtered. For filtration of the collected sample, 2 mL of distilled water, 1 mL of lung bleach-digestion solution, and 2 mL of distilled water were added, in that order. The samples were filtered using a PC filter (25-mm diameter, 0.2-µm pore size) to produce sample fragments for transmission electron microscopy (TEM) analyses (Fig. 4).

The filter was coated with carbon using a sample-coating machine, fixed on a grid, melted using the Jaffe washer containing the chloroform solution, and dried to make a grid for analysis. Analysis was performed using × 1,200–20,000 magnification at 300 open grids per sample. These conditions were set in consideration of the lowest sensitivity reported in previous studies^18–23^. The counting standard for analysis of length was > 0.5 µm; diameter, > 0.01 µm; and aspect ratio, > 3. The crystal structure of asbestos in 300 open grids was analyzed using selected area electron diffraction, and a chemical component analysis was performed using TEM (H-7650, HITACHI) and an energy-dispersive X-ray spectrometer. The asbestos concentration was calculated using the following equation:

$$\text{C }\left(\text{fiber}/\text{dry g}\right)=\frac{Effective\, area \left({mm}^{2}\right)\times \left(\frac{No.\,of\,asbestos\, fibers}{No.\, of\, open\, grids}\right)}{Open\, grid\, area\, \left({mm}^{2}\right)\times\, sample\, weight \left(g\right)\times (D/W)}$$

where an effective area (mm^2^) is the effective area sampled on the filter, sample weight is the weight of the wet lung cell sample (g), and D/W is the ratio of the weight after the drying process to the weight of the wet sample.

### Statistical analysis

All statistical analyses were performed using the SAS 9.4 software (SAS Institute Inc., Cary, NC, USA). Student’s t-test was performed to compare the asbestos burden by sex and morphological characteristics (asbestos type and length, diameter, and aspect ratio). An analysis of variance followed by the Scheffe test was conducted to compare the asbestos burden among the three groups: male LCPs environmentally exposed to asbestos (EA-LCP), female EA-LCPs, and male patients occupationally exposed to asbestos (OA-LCP) (no female patient occupationally exposed to asbestos answered the questionnaire). Multiple regression analysis was performed to identify factors affecting asbestos burden in the lung tissue of the patients. Results with p-values below 0.05 were considered statistically significant.
